# Integrated bioinformatic analysis reveals the underlying mitochondria-associated endoplasmic reticulum membranes-related biomarkers for atrial fibrillation

**DOI:** 10.3389/fphys.2025.1647275

**Published:** 2025-09-09

**Authors:** Youcheng Wang, Mengyang Song, Huanting Liu, Sini Fang, Yumeng Lei, Jiulin Liu, Jiayuan Zhang, Ke Zhang, Ying Mao, Liqiu Yan

**Affiliations:** ^1^ Department of Cardiology, The Affiliated Dongguan Songshan Lake Central Hospital, Guangdong Medical University, Dongguan, Guangdong, China; ^2^ Dongguan Key Laboratory of Cardiovascular Aging and Myocardial Regeneration, Dongguan Cardiovascular Research Institute, Dongguan, China; ^3^ School of Medicine, Wuhan University of Science and Technology, Wuhan, China

**Keywords:** atrial fibrillation, mitochondria-associated endoplasmic reticulum membranes, biomarker, immune infiltration, bioinformactics analysis

## Abstract

**Purpose:**

To provide novel insights into the diagnosis of atrial fibrillation (AF), we aimed to identify mitochondria-associated endoplasmic reticulum membranes (MAMs)-related biomarkers for AF.

**Methods:**

The training and validation datasets of AF were sourced from the Gene Expression Omnibus (GEO) database. A comprehensive analysis was conducted to identify MAM-related biomarkers, including support vector machine-recursive feature elimination (SVM-RFE) and differentially expressed analysis. Moreover, causal effects of biomarkers on AF were assessed through the two-sample Mendelian randomization (MR) analysis. Functional enrichment, immune infiltration, and single-cell analyses were conducted to investigate the possible mechanisms of biomarkers regulating AF. Finally, the expression of biomarkers was validated at the mRNA and protein levels by developing an *in-vivo* canine AF model.

**Results:**

Through the comprehensive analysis, TP53, HLA-G, and MAPKAPK5 were identified, which were highly expressed in atrial tissues of AF samples. Notably, MAPKAPK5 was a risk factor for occurrence of AF (*P* = 0.022, OR = 1.065, 95%CI = 1.009–1.125). Enrichment analysis revealed that three biomarkers were associated with immune-related pathways. Immune infiltration further demonstrated that a total of infiltration abundance of 18 immune cells was significantly different between AF and controls, and all biomarkers had marked positive associations with these immune cells. Moreover, at the cellular level, the expression of TP53 and MAPKAPK5 was markedly different in lymphoid cells and neutrophils between AF and controls. At the experimental levels, the expression of three biomarkers was significantly higher in the AF model than that in the control model, consistent with the bioinformatics results.

**Conclusion:**

We identified three potential MAMs-related biomarkers (TP53, HLA-G, and MAPKAPK5) for AF, thereby providing novel insights for the prevention and treatment of AF.

## Introduction

Atrial fibrillation (AF) is one of the most prevalent arrhythmias, accounting for approximately one-third of all hospitalizations due to arrhythmias. Epidemiological studies indicate that the global prevalence of AF ranges from 1% to 2%, increasing gradually with age (Brundel et al., 2022). AF can lead to reduced cardiac and cognitive function, and an elevated risk of stroke and other thromboembolic events, all of which substantially contribute to increased mortality and disability rates. Clinically, most AF patients progress from initial paroxysmal to persistent AF, and ultimately to permanent AF (de Vos et al., 2010). The underlying pathophysiological mechanisms are complex and remain incompletely understood. Despite various available treatments, including pharmacotherapy, catheter ablation, and surgical interventions, the outcomes are often unsatisfactory, particularly in patients with persistent atrial fibrillation, who exhibit a high long-term recurrence rate (Bosch et al., 2018). Therefore, further elucidation of the pathogenesis of AF is essential for enhancing early diagnosis and developing personalized treatment strategies for affected patients.

The mitochondria-associated endoplasmic reticulum membranes (MAMs) are a dynamic membrane structure formed between the endoplasmic reticulum (ER) and the mitochondrial membranes through a series of protein connections. This structure serves not only as a physical contact point between the two organelles but also as a platform for material exchange and signal transmission, participating in various physiological and pathological processes such as lipid metabolism, calcium signaling pathways, apoptosis, and autophagy in cells (Luan et al., 2021; Luan et al., 2022). In recent years, emerging evidence has indicated that MAMs-related proteins play significant roles in various cardiovascular diseases. FUNDC1, a mitogenic receptor, is enriched at the contact sites between mitochondria and the ER, facilitating the formation of MAMs, thereby modulating cytosolic Ca^2+^ homeostasis and mitochondrial dynamics, and averting cardiac dysfunction (Li et al., 2021). SUMOylation of Drp1 (a dynamin-related GTPase that critically mediates fission) enhances mitochondrial autophagy during reperfusion, thereby preventing reactive oxygen species (ROS), myocardial apoptosis, and myocardial injury (Tong et al., 2020). However, little research has yet addressed the relationship between MAMs and AF.

Mendelian randomization (MR) has arisen as a robust analytical method for deducing causal links between exposures and outcomes by utilizing genetic variants as instrumental variables, a design that is less prone to confounding and reverse causation biases. This methodology signifies a progressive advancement in causal inference, enhancing observational studies—such as those employing extensive databases with weighted regression, subgroup analyses, and multivariate adjustments—that initially discern potential associations and establish the foundation for causal hypotheses (Guo et al., 2024b). Observational studies function as essential preliminary frameworks by identifying potential links, whereas MR serves as a rigorous subsequent tool to confirm causality, addressing challenges such as residual confounding and reverse causation that sometimes obscure observational results. This methodological advancement has been extensively utilized to analyze causative relationships in intricate disorders, demonstrating efficacy in clarifying disease mechanisms (Guo and He, 2024; Xu et al., 2025b). Building on such methodological advancements, this study aims to investigate the connection between MAMs-related genes (MAMRGs) and AF through bioinformatics analysis, as well as to assess the causal relationship between these genes and the onset of AF using MR analysis, thereby strengthening the rationale. Additionally, we conduct functional enrichment analysis, immune infiltration analysis, drug prediction, and single-cell analysis to preliminarily explore the potential functional pathways and pathogenesis associated with these biomarkers. This comprehensive approach aims to enhance our understanding of the molecular mechanisms underlying AF and provide new insights into its clinical diagnosis and treatment.

## Materials and methods

### Data acquisition and preprocessing

Gene expression datasets (GSE14975, GSE41177, and GSE79768) for AF were retrieved from the Gene Expression Omnibus (GEO) database (https://www.ncbi.nlm.nih.gov/geo/). The transcriptional profiling in GSE14975 (Platform: GPL570) comprises left atrial myocardium from 5 AF patients and 5 matched samples of patients in sinus rhythm (controls) (Adam et al., 2010). GSE41177 (Platform: GPL570) consists of 19 left atrial appendage samples from 16 AF patients and 3 controls (Yeh et al., 2013). These two datasets were merged (the clinical information of these two datasets was displayed in Supplementary Table 1), yielding a training set of 21 AF cases and 8 controls, after which the “sva” package (Leek et al., 2012) was employed to eliminate batch effects, hence ensuring data uniformity. GSE79768 (Platform: GPL570) was the validation set in this study, which included 13 left atrial samples from 7 AF patients and 6 controls (Tsai et al., 2016). Besides, a total of 68 mitochondria-associated ER membrane-related genes (MAMRGs) were extracted from the early research (Li H. M. et al., 2024).

### Identification of AF-related and MAMs-related genes in AF (AF-MAMRGs)

To identify AF-related genes, the “limma” R package (version 3.54.0) was employed to discover the differentially expressed genes (DEGs) between AF and controls in the training set (Ritchie et al., 2015). The |logFoldChange| > 1 and adjusted *P*-value <0.05 were screening thresholds. Moreover, results were displayed as a volcano plot [using the “ggplot” R package (version 3.4.1) (Wickham, 2016)] and a heatmap [using the “ComplexHeatmap” R package (version 2.14.0) (Gu et al., 2016)]. Subsequently, AF-MAMRGs were determined through utilizing a Venn diagram to link the AF-related genes and MAMRGs. The STRING database (https://cn.string-db.org/) was applied to construct the protein-protein interaction (PPI) network to explore the direct and indirect associations among the AF-MAMRGs (parameter setting: Low confidence = 0.4).

### Functional enrichment analysis

To comprehend the potential biological functions of AF-MRMRGs, the functional enrichment analysis was conducted with the “clusterProfiler” R package (version 4.2.2), including Gene Ontology (GO) and Kyoto Encyclopedia of Genes and Genomes (KEGG) analyses (*P* < 0.05) (Wu et al., 2021). GO analysis comprised three components: Biological Process (BP), Cellular Component (CC), and Molecular Function (MF).

### Identification of MAMs-related biomarkers for AF

The support vector machine-recursive feature elimination (SVM-RFE) is a technique for feature selection that recursively eliminates the least important features using SVM. To evaluate the significance of AF-MRMRGs in the diagnosis of AF, SVM-RFE was first utilized to screen characteristic genes by the “e1071” R package. Simultaneously, a least absolute shrinkage and selection operator (LASSO) regression model with 10-fold cross-validation was constructed using the glmnet R package (v4.1.7) to enhance confidence in the identified genes (Huang et al., 2024). After that, the expression of characteristic genes between AF and controls was analyzed in both training and validation sets, which with the significant alterations and consistent expression trends in two sets were denoted as biomarkers for AF.

### Construction and assessment of a nomogram

A nomogram is a graphical tool widely used to predict the probability of a particular outcome based on a set of variables. Therefore, based on the expression levels of the identified biomarkers, a nomogram was developed to predict the probability of AF. Moreover, the calibration curve, receiver operating characteristic (ROC) curve, and decision curve analysis (DCA) curve were plotted to assess the predictive performance of the nomogram.

### Gene set enrichment analysis (GSEA) and gene set variation analysis (GSVA)

The GSEA (Guo et al., 2024a; Xu et al., 2025a; Yang et al., 2025) and GSVA analyses were conducted to investigate the potential mechanisms of biomarkers in the occurrence of AF. For GSEA, the “c2. cp.kegg.v7.4. symbols.gmt” was extracted from the MSigDB database (https://www.gsea-msigdb.org/gsea/msigdb) as the reference set. The correlations between biomarkers and all genes were calculated, and correlation coefficients were ranked. GSEA was carried out by the “clusterProfiler” R package (version 4.2.2) with a *P*-value <0.05. Additionally, the samples in the training set were categorized into high- and low-expression groups according to the expression of each biomarker. With “h.all.v7.4. symbols.gmt” downloaded from the MSigDB database as the reference set, the GSVA score of each pathway in two expression groups was calculated by the “GSVA” R package (Li J. et al., 2024), and the differences in GSVA scores between the two groups were assessed via the “limma” R package with |t| > 2 and *P*-value <0.05.

### Immune infiltration

Previous studies have demonstrated that the immune system undergoes significant changes during AF and interacts with the environment and cells involved in the initiation and maintenance of AF (Yao et al., 2022). Consequently, the infiltration abundance of 28 immune cells in AF and controls was evaluated by single-sample gene set enrichment analysis (ssGSEA) (Cui et al., 2025), and the differences between AF and controls were analyzed via the Wilcoxon test (*P* < 0.05). To further explore the correlations between biomarkers and immune infiltration, the Spearman algorithm was applied.

### Prediction of potential drugs for AF treatment

The DGIdb database (https://www.dgidb.org/) (Cannon et al., 2024) is an online database of drug-gene interactions, with data sourced from multiple drug databases (DrugBank, PharmGKB, ChEMBL), clinical trial databases, and PubMed literature. The potential drugs were predicted for AF using biomarkers as the keywords. To further investigate the specific mechanism, molecular docking was conducted by AutoDock Vina (Eberhardt et al., 2021). The 3D structures of drugs were downloaded from the PubChem database (SDF file) (Kim et al., 2025), and the SDF files were transferred into PDB files via Babel GUI(O'Boyle et al., 2011). Moreover, the 3D structures of proteins were extracted from the Protein Data Bank Database (Burley et al., 2025). Finally, the PyMol software (Mooers and Brown, 2021) was employed to view and visualize the results.

### Collection and analysis of the single-cell RNA sequence (scRNA-seq) data

The scRNA-seq dataset of AF (GSE224959, GPL18573) was also retrieved from the GEO database, collecting left atrial tissue from 7 AF samples and 5 controls (Hulsmans et al., 2023). The processing of data was performed via the “Seurat” R package (Satija et al., 2015) according to the following criteria: (1) cells were removed with less than 200 genes expressed; (2) genes were excluded with less than 3 cells covered; (3) cells were excluded with more than 20% mitochondrial genes; (4) cells were removed with 200 ≤ gene numbers ≤3,000; and (5) genes were excluded with 200 ≤ count numbers ≤10,000. After identification of the top 2,000 highly variable genes and PCA dimensionality reduction analysis, the cluster analysis was conducted for remaining cells using the FindNeighbors and FindCluster functions (Di et al., 2022) in the “Seurat” R package. Subsequently, cell clusters were annotated through comparing the differentially expressed genes of each cluster with the marker genes of each cell type in the CellMarker database (Zhang et al., 2019). Afterwards, the percentage of each cell type was counted, and the expression of each biomarker in each cell type between AF cases and controls was measured. Moreover, the pseudotime analysis of the cell type where the expression of biomarkers between AF and controls was at significant levels was conducted to investigate the change of biomarker expression during cell differentiation. Finally, to reveal interactions among these cell types, CellPhoneDB (Efremova et al., 2020) was employed.

### Mendelian randomization (MR) analysis

To further investigate the causal effect of biomarkers on AF, MR analysis was carried out using TwoSampleMR R package (Hemani et al., 2018). The data of the exposure and outcome were sourced from the IEU OpenGWAS database, including eqtl-a-ENSG00000089022 (MAPKAPK5) (31,470 samples and 15,599 single nucleotide polymorphisms (SNPs)), eqtl-a-ENSG00000141510 (TP53) (31,684 samples and 18,457 SNPs), eqtl-a-ENSG00000204632 (HLA-G) (25,690 samples and 28,777 SNPs), and bbj-a-71 (AF) (36,792 samples (8,180 AF and 28,612 control) and 5,018,048 SNPs). To satisfy the three assumptions of MR analysis, SNPs employed as IVs must meet the following criteria: (1) IVs must be significantly relevant to the exposure with P < 5 × 10^−6^; (2) the SNPs with linkage disequilibrium (LD) were removed (parameter settings: *r* ^2^ = 0.01, kb = 100); (3) the F statistic value was calculated to evaluate the strength of SNPs, and those with F less than 10 were excluded. Afterwards, the two-sample MR analysis was conducted through combining the harmonise_data and mr functions with five methods (Weighted median, MR Egger, Simple mode, Inverse variance weighted (IVW), and Weighted mode), and among these methods, the IVW was the main method. Causal effects were represented by odds ratios (OR) and their 95% confidence intervals (CIs), and statistical significance was defined as *P* less than 0.05. Furthermore, to measure the robustness of results, the sensitivity analysis was conducted through the heterogeneity test, horizontal pleiotropy test, and leave-one-out (LOO) test. The choice between IVW-fixed effects (IVW-FE) and IVW-random effects (IVW-RE) was determined by the presence of heterogeneity. Specifically, IVW-RE was selected when heterogeneity existed, whereas IVW-FE was adopted in the absence of such heterogeneity. The mr_heterogeneity () function was employed to detect heterogeneity. Horizontal pleiotropy was identified using the MR-Egger intercept method: a significant difference between the intercept term of MR-Egger regression and 0 (P < 0.05) indicated the existence of horizontal pleiotropy. Additionally, the LOO method was used to evaluate the impact on the MR results after excluding each individual SNP.

### AF model establishing and electrophysiological measurement

The AF model was established by rapid atrial pacing using canines. The sham group received pacemaker implantation procedure under sterile conditions without atrial pacing. The pacing group underwent pacemaker implantation with continuous rapid atrial pacing (450 beats/min) for 2 months before the electrophysiological measurements. All electrophysiological measurements were recorded in a computerized electrophysiology system (Lead 7,000, Jinjiang Inc., China). An S1S1 programmed stimulus method (with cycle length of 120 ms, 100 ms, and 75 ms; 5 s each, performed 3 times per frequency) was used to evaluate AF durations. AF was characterized as an irregular atrial rate exceeding 500 bpm and that lasts for more than 5 s.

All trial protocols were approved by the Laboratory Animal Welfare & Ethics Committee of Dongguan Songshan Lake Central Hospital. The experiments involving live vertebrates were carried out in strict accordance with the relevant guidelines and regulations. All methods were reported in accordance with ARRIVE guidelines.

### Quantitative real-time polymerase chain reaction (qRT-PCR)

Total RNA was isolated from canine atrial tissue samples utilizing RNA extraction solution (G3013, Saiwei Biotechnology, CN) in accordance with the procedure. Tissue homogenization was conducted using a three-dimensional cryogenic grinder (KZ-5F-3D, Saiwei Biotechnology, CN), followed by purification processes involving chloroform extraction, isopropanol precipitation, and washing with 75% ethanol solution. The purity and concentration of RNA were evaluated using a Nanodrop 2000, and the concentration was standardized to 200 ng/μL cDNA synthesis was performed utilizing SweScript All-in-One RT SuperMix for qPCR (One-Step gDNA Remover, G3337, Saiwei Biotechnology, CN) to reverse transcribe RNA into cDNA, with the reaction protocol established at 25 °C for 5 min, 42 °C for 30 min, and 85 °C for 5 s. The 2×Universal Blue SYBR Green qPCR Master Mix (G3326, Saiwei Biotechnology, CN) was utilized for qPCR, with each 15 μL reaction comprising the master mix, primer mixs for HLA-G, TP53, and MAPKAPK5, cDNA template, and nuclease-free water. Primer sequences are presented in Table 1. Each sample was run in triplicate. The amplification was performed on a CFX Connect Real-Time PCR System (CFX Connect, Bio-Rad, CN) with the thermal cycling conditions: initial denaturation at 95 °C for 30 s, followed by 40 cycles of 95 °C for 15 s and 60 °C for 30 s, and a melting curve stage from 65 °C to 95 °C. Relative gene expression levels were calculated using the 2^(−ΔΔCT)^ method.

**TABLE 1:** The primer sequences of biomarkers.

Gene	Primer sequences (5′-3′)
GAPDH-S	GGGTGATGCTGGTGCTGAGTAT
GAPDH-A	TTGCTGACAATCTTGAGGGAGTT
HLA-G	ACTGCAGAGCACGATAGGAAC
HLA-G	GATCTCCGCAGGGTAGAAGC
TP53-S	CTGAGGAGGAGAATTTCCACAAG
TP53-A	CTTCAGCTCCAAGGCTTCATTC
MAPKAPK5-S	GAAATCTGGCATCATACCTACCTC
MAPKAPK5-A	CCACTCTTCTTCTGGGAACTCAA

### Western blotting

The expression levels of TP53, MAPKAPK5, and HLA-G in atria were detected by Western blotting. After atrial tissues were washed with PBS, lysis buffer prepared with proteinase inhibitors and RIPA strong lysis buffer was added. Tissues were homogenized on ice for 30 min using a liquid nitrogen grinder. Samples were centrifuged at 4 °C, 12,000 rpm for 20 min using a refrigerated high-speed centrifuge. After centrifugation, collect the supernatant and measure the protein concentration using the BSA kit. An appropriate amount of 5× loading buffer was added to the remaining supernatant, and heat at 95 °C in a metal bath for 10 min. Electrophoresis of protein samples was performed using sodium dodecyl sulfate polyacrylamide gel electrophoresis (SDS-PAGE). After electrophoresis, proteins were transferred onto a PVDF membrane and blocked with 5% non-fat dry milk for 2 h. After blocking, the membrane was washed 5 times with TBST solution, each for 6 min. The membrane was then incubated with primary antibodies: anti-TP53 (IPD-ANP1072, IPODIX, China, dilution 1:500), anti-MAPKAPK5 (IPD-ANP9498, IPODIX, China, dilution 1:500), anti-HLA-G (GB115645, Servicebio, China, dilution 1:500), and anti-GAPDH (GB15004, Servicebio, China, dilution 1:10,000) at 4 °C for at least 12 h. After the blocking period, wash the membrane 5 times with TBST, each for 6 min. Next, add the corresponding secondary antibody working solution and incubate for 2 h at room temperature. After incubation, wash the membrane 3 times with TBST, each for 6 min. To ensure that the loaded samples were of equal concentration, the ratio of band intensity to GAPDH was calculated to quantify the relative expression levels of these proteins.

### Statistical analysis

All statistical analyses were conducted employing R software (version 4.2.2). The disparities were analyzed via the Wilcoxon test (n = 2). The data obeyed normal distribution characteristics and are shown as the mean ± standard deviation (SD). Two-sample independent Student’s t-test was used to compare the means of two groups. A statistically significant *P-*value is less than 0.05.

## Results

### Identification and enrichment analysis of AF-MAMRGs

**FIGURE 1:**
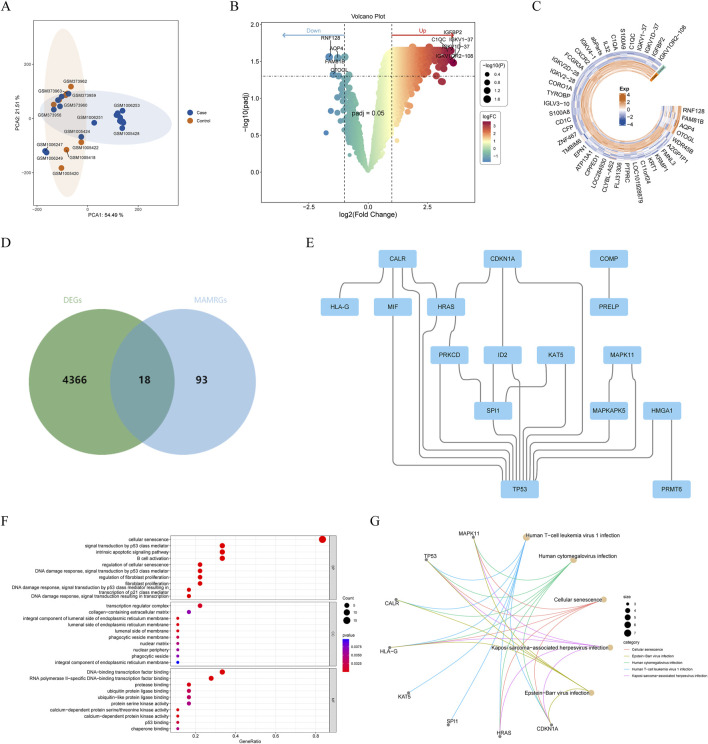
Identification and enrichment analysis of atrial fibrillation (AF)-related and mitochondria-associated endoplasmic reticulum membrane (MAM)-related genes (AF-MAMRGs) **(A)** Scatter plot suggesting principal component analysis (PCA) distribution for each sample after batch correction **(B,C)** The differentially expressed genes (DEGs) between AF and controls **(B)** The valcano plot **(C)** The heatmap **(D)** Venn diagram showing the overlap of genes between DEGs and MAMRGs **(E)** The protein-protein interaction (PPI) network of AF-MAMRGs **(F,G)** Functional enrichment analysis **(F)** Gene ontology (GO) **(G)** Kyoto Encyclopedia of Gene and Genomes (KEGG).

After removing the batch effects, PCA demonstrated that AF samples could be clearly separated from control samples (Figure 1A). Through differentially expressed analysis, a total of 4,384 DEGs between AF and controls were obtained, including 4,380 upregulated and 4 downregulated genes (Figures 1B,C). Subsequently, through combination of MAMRGs and DEGs, there were 18 AF-MAMRGs obtained (Figure 1D). The PPI network of AF-MAMRGs included 16 nodes and 23 edges (Figure 1E), with TP53 had interactions with most proteins, such as MIF, MAPKAPK5, and SPI1. Additionally, the enrichment analysis illustrated that a total of 958 GO items and 75 KEGG pathways were markedly enriched (Supplementary Table 2). The top 10 GO items and the top 5 KEGG pathways were displayed, including “cellular senescence (GO-BP item),” “integral component of lumenal side of endoplasmic reticulum membrane (GO-CC item),” “DNA-binding transcription factor binding (GO-MF item),” and “Human T-cell leukemia virus 1 infection (KEGG)” (Figures 1F,G).

### Determination of MAMs-related biomarkers for AF

**FIGURE 2:**
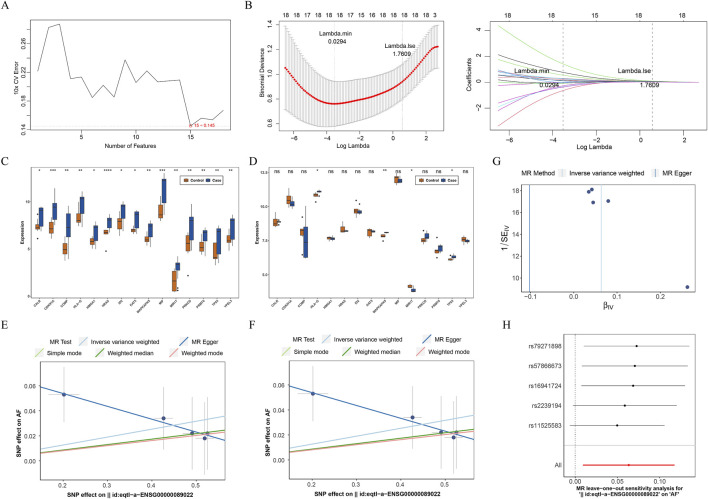
Identification of MAM-related biomarkers for AF **(A)** Selection of characteristic genes based on the support vector machine recursive feature elimination (SVM-RFE) algorithm **(B)** Selection of characteristic genes based on the least absolute shrinkage and selection operator (LASSO) regression algorithm **(C,D)** The expression of 15 characteristic genes in AF and controls in training and validation sets **(C)** The training set **(D)** The validation set **(E–H)** The causal effect of MAPKAPK5 on AF **(E)** The scatter plot **(F)** The forest plot **(G)** The funnel plot **(H)** The leave-out-out (LOO) method ns: not significance *P < 0.05, **P < 0.01, ***P < 0.001, ****P < 0.0001.

To discover the genes with diagnostic value for AF among AF-MAMRGs, we first identified 15 characteristic genes through SVM-RFE and these 15 genes were incorporated in the LASSO results (17 genes) to ensure robustness (Figures 2A,B). For the expression examination, in both training and validation sets, TP53, MAPKAPK5, and HLA-G were significantly upregulated in AF compared to controls (Figures 2C,D).

Consequently, these three genes were identified as biomarkers for AF. To further analyze the causal effect of biomarkers on AF, a two-sample MR analysis was conducted. Since the number of SNPs of TP53 and HLA-G were less than 3 after IV selection, only the causal effect of MAPKAPK5 on AF was measured. Results illustrated that MAPKAPK5 had a significant causal association with AF (*P* = 0.022) and was a risk factor for AF onset (OR = 1.065, 95%CI = 1.009–1.125) (Table 2). Moreover, the scatter plot, forest plot, and funnel plot also demonstrated the consistency and reliability of the findings (Figures 2E–G). The Q statistic P-values for MR in this analysis were all below 0.05, which prompted the selection of a fixed-effect model. No heterogeneity and horizontal pleiotropy was detected (Q value = 0.415; Egger_intercept = 0.074; *P* = 0.143), and removal of any individual SNP had no effect on results, suggesting that the results were reliable and robust (Table 3; Figure 2H).

**TABLE 2:** MR analysis.

Exposure	Outcome	Method	nsnp	b	se	P-value	OR	or-lci95	or-uci95
MAPKAPK5|| id:eqtl-a-ENSG00000089022	AF||id:bbj-a-71	MR Egger	5	−0.103	0.089	0.33	0.902	0.759	1.073
		Weighted median	5	0.043	0.032	0.179	1.044	0.98	1.113
		Inverse variance weighted (fixed effects)	5	0.063	0.028	0.022	1.065	1.009	1.125
		Simple mode	5	0.041	0.036	0.328	1.041	0.97	1.119
		Weighted mode	5	0.041	0.035	0.311	1.041	0.972	1.116

nsnp, number of single nucleotide polymorphism; se, standard error; OR, odd ratio; CI, confidence interval.

**TABLE 3:** Heterogeneity and pleiotropy analyses.

Outcome	Exposure	Heterogeneity				Pleiotropy		
		Method	Q	Q_df	Q_pval	Egger_ intercept	se	Pval
AF||id:bbj-a-71	MAPKAPK5|| id:eqtl-a-ENSG00000089022	MR Egger	0.038	3	0.998	0.074	0.038	0.143
		Inverse variance weighted	3.934	4	0.415			

Q, Cochran’s Q test estimate; Q_df, Q_degree of freedom; Q_pval, significance; se, standard error.

### Development of a nomogram with predicting efficacy for AF

**FIGURE 3:**
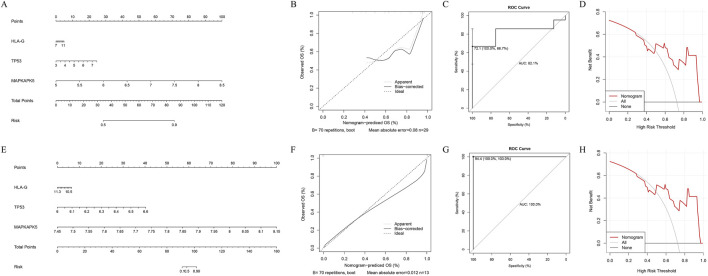
Development and assessment of the nomogram. The four figures in the upper row represent the results of the training set, while the figures in the lower row correspond to the results of the validation set. **(A–E)** The visualization of the nomogram with diagnostic value for AF patients **(B–H)** The assessment of the nomogram **(B,F)** The calibration curve **(C,G)** The receiver operating characteristic (ROC) curve **(D,H)** The decision curve analysis (DCA).

We enhanced the nomogram in both training and validation cohorts to forecast the occurrence of AF based on the expression of three biomarkers (Figures 3A,E). Both calibration curves indicated that the slopes were approximately 1 (Figures 3B,F). The area under the ROC curves were 0.821 and 1, respectively (Figures 3C,G). Furthermore, the DCA indicated that the nomogram exhibited the highest net benefit (Figures 3D,H). These results indicated that the nomogram had high accuracy and robustness for predicting AF.

### Enrichment analyses for biomarkers

**FIGURE 4:**
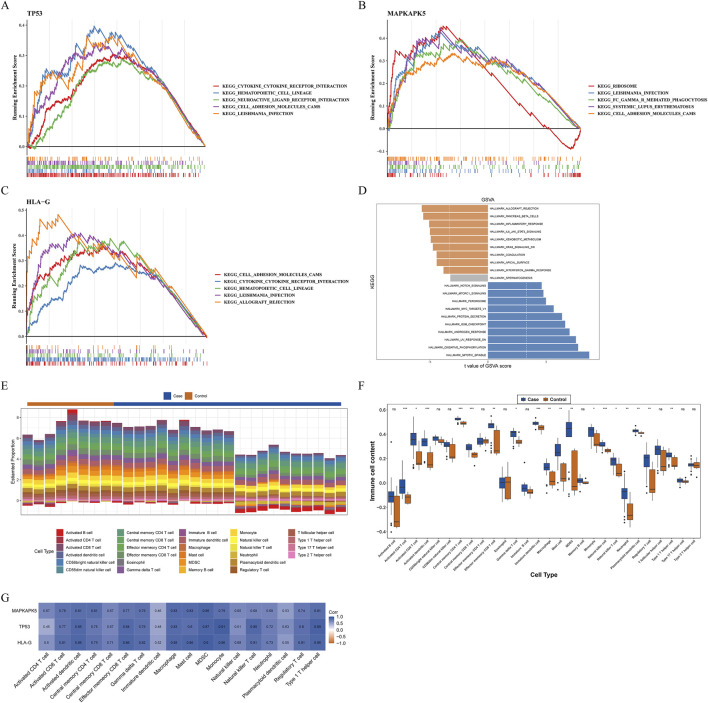
Exploration of possible mechanisms by which biomarkers modulate AF **(A–D)** Functional enrichment analysis **(A–C)** Gene set enrichment analysis (GSEA) **(A)** TP53 **(B)** MAPKAPK5 **(C)** HLA-G **(D)** Gene set variation analysis (GSVA) for biomarkers **(E–G)** Immune infiltration analysis **(E)** The percentage of infiltration abundance of each immune cell in AF and controls **(F)** The differences in infiltration abundance of each immune cell between AF and controls **(G)** Correlations between biomarkers and immune cells with significant differences in infiltration abundance ns: not significance *P < 0.05, **P < 0.01, ***P < 0.001, ****P < 0.0001.

Next, we analyzed the specific signaling pathways involved in the three biomarkers and explored the impact of the biomarkers on the signaling pathways associated with AF progression. GSEA results demonstrated that three biomarkers were involved in immune-related pathways, such as “cytokine-cytokine receptor interaction,” “chemokine signaling pathway,” “TOLL like receptor signaling pathway,” “MAPK signaling pathway,” “natural killer cell mediated cytotoxicity” (Figures 4A–C) (Supplementary Table 3). Moreover, these biomarkers were also involved in metabolism-related pathways, including “glycerophospholipid metabolism” and “propanoate metabolism” (Supplementary Table 3). Results of GSVA illustrated that the HALLMARK pathways enriched of the two expression groups of TP53 and HLA-G were identical. The high-expression group was enriched in “mitotic spindle,” “oxidative phosphorylation,” “UV response DN” and other 13 pathways (Figure 4D; Supplementary Table 3). The low-expression group was enriched in “allograft rejection,” “inflammatory response,” “IL6-JAK-STAT3 signaling” and other 6 pathways (Figure 4D; Supplementary Table 3).

### Assessment of immune infiltration

Previous studies have demonstrated that the immune response may play an important role in the development and maintenance of AF. Moreover, results of GSEA revealed that three biomarkers were relevant to immune-related pathways in our study. Therefore, we further measured the infiltration abundance of 28 immune cells in AF and controls, and group differences were analyzed (Figures 4E,F). Between AF and control groups, the infiltration abundance of 18 immune cells was considerably increased in the AF group compared to the control group, including macrophages, activated dendritic cells, neutrophils, monocytes, etc.(Figure 4F). Both activated CD4^+^/CD8^+^ T cells and central memory CD4^+^/CD8^+^ T cells exhibited significant differences in infiltration levels between groups, highlighting the involvement of diverse T cell subsets. Moreover, all biomarkers had marked positive relevance to these immune cells, and the highest correlation was between TP53 and monocytes (cor = 0.91, *P* = 7.495e-12), meanwhile, myeloid-derived suppressor cells (MDSC) was highly correlated with all biomarkers (cor >0.95) (Figure 4G).

### Drug prediction and molecular docking simulation

**FIGURE 5:**
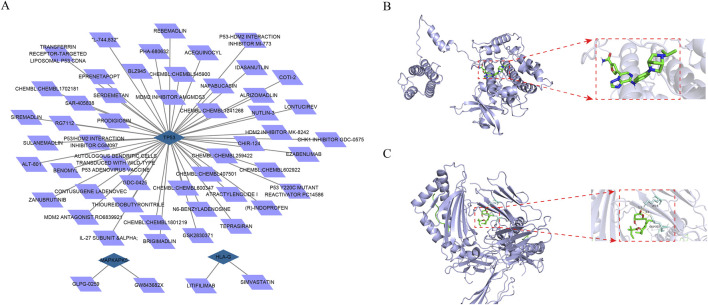
Prediction of drugs with interacting with biomarkers **(A)** The drug-mRNA nework Blue represents mRNAs and purple represents drugs **(B,C)** The molecular docking between biomarkers and drug with the highest interaction score **(B)** The molecular docking between GLPG-0259 and MAPKAPK5 **(C)** The molecular docking between SIMVASTATIN and HLA-G.

To find drugs for AF therapy, we searched the DGIdb database. Results demonstrated that two potential drugs interacted with HLA-G, two potential drugs interacted with MAPKAPK5, and 49 potential drugs interacted with TP53 (Figure 5A). Moreover, according to the ranking of interaction scores, the drug with the highest interaction score for each biomarker was selected for molecular docking. Results demonstrated that the docking energy between MAPKAPK5 and GLPG-0259 was −9.5 kcal/mol, and SIMVASTATIN had a strong binding affinity with residues LYS-6 and GLU-232 of HLA-G through hydrogen bonding, with a docking energy of −5.74 kcal/mol (Figures 5B,C). These results indicate a stable binding capacity between MAPKAPK5 and HLA-G and their respective targeted drugs, suggesting their potential as therapeutic candidates for further investigation in related pathways. However, the docking energy between TP53 and THIOUREIDOBUTYRONITRILE was −3.84 kcal/mol, indicating that the bond between them was not stable.

### Expression of biomarkers at the single-cell level

**FIGURE 6:**
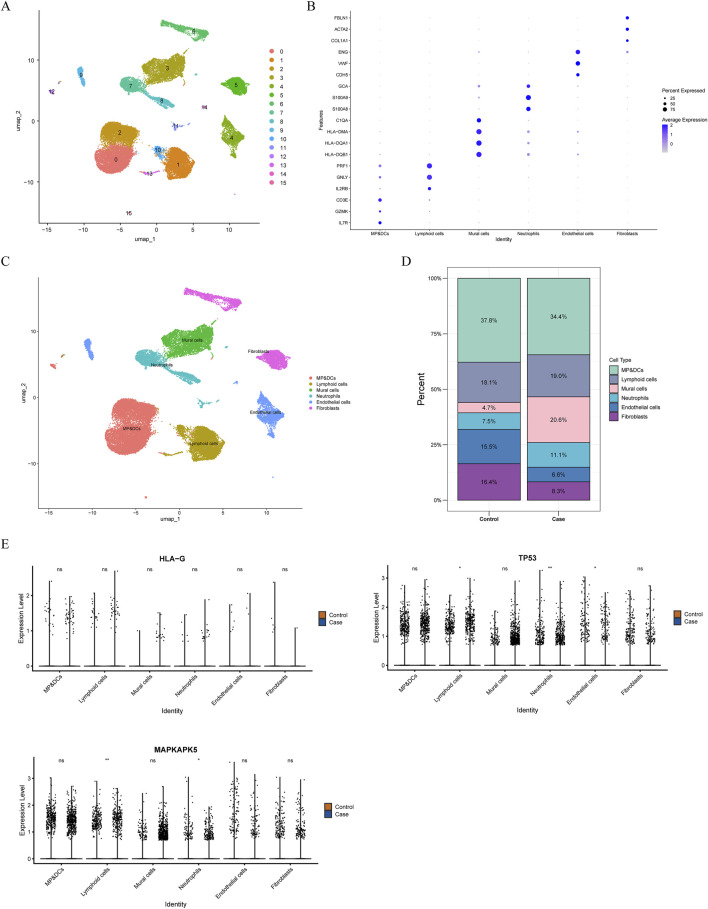
Single-cell analysis **(A)** UMAP distribution of 16 independent clusters **(B)** Bubble diagram shows the expression of marker genes in each annotated cell types **(C)** UMAP distribution of 6 cell types **(D)** The proportions of each cell type **(E)** The expression of biomarkers in each cell type in AF and controls From top to bottom: HLA-G; TP53; MAPKAPK5.

**FIGURE 7:**
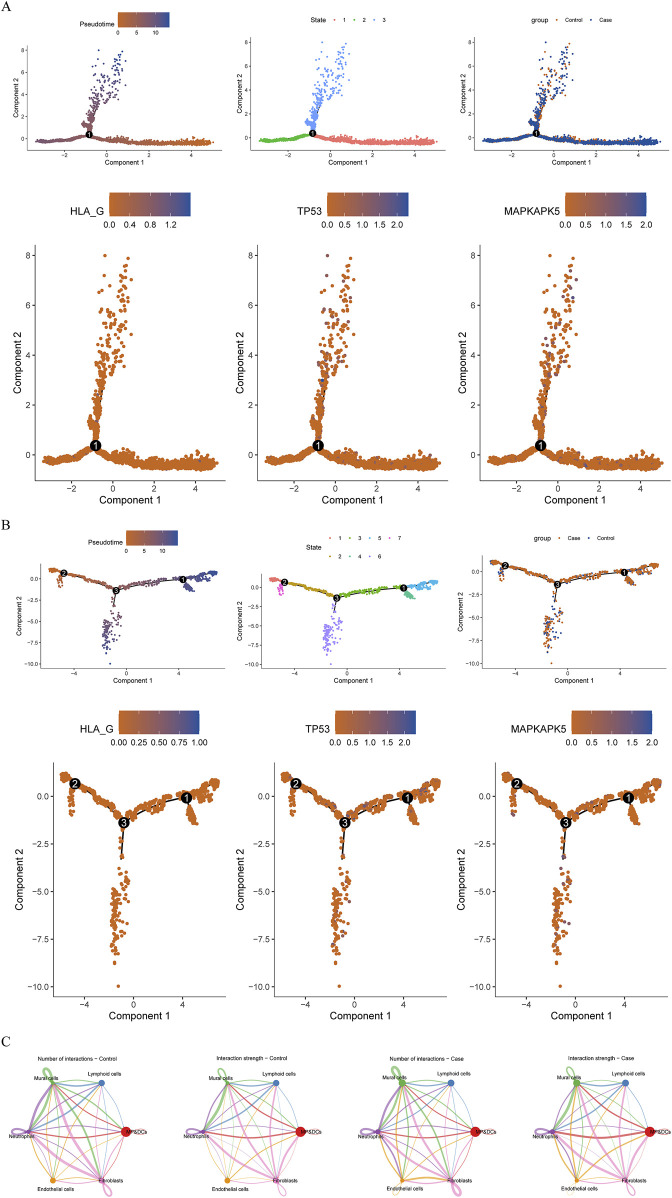
Pseudotime analysis and cell communication analyses **(A,B)** Pseudotime analysis **(A)** Lymphoid cells **(B)** Neutrophils From left to right: The cells are coloured according to pseudotime value; The cells are coloured according to cell states; The cells are coloured according to different samples; The cells are coloured according to the expression of biomarkers **(C)** Cellular communication network illustrating the number and strength of interactions.

Following quality control of scRNA-seq data, the top 2,000 highly variable genes were determined, and the top 30 principal components (PCs) were selected (Supplementary Figure S1). Through cluster analysis, a total of 16 cell clusters were determined, which were annotated as 6 cell types: mononuclear phagocytes and dendritic cells (MP/DCs), lymphoid cells, mural cells, neutrophils, endothelial cells, and fibroblasts (Figures 6A–C). Among these cell types, MP/DCs accounted for the highest proportion in both AF and controls (Figure 6D). Expression analysis illustrated that between AF and controls, the expression of TP53 in lymphoid cells, neutrophils, and endothelial cells and the expression of MAPKAPK5 in lymphoid cells and neutrophils were significantly different (Figure 6E). However, between AF and control groups, the expression of HLA-G in each cell type did not differ significantly (Figure 6E). Afterwards, the pseudotime analysis was employed on lymphoid cells and neutrophils. Results demonstrated that TP53 and MAPKAPK5 were expressed throughout cell differentiation (Figures 7A,B). Additionally, cell-cell interaction analysis illustrated communication patterns among 6 cell types in the AF microenvironment. Compared to the control group, the bidirectional interaction between neutrophils and endothelial cells in AF was increased (Figure 7C), which thus drew our focus. Further analysis of ligand-receptor pairs revealed an increased signaling intensity of the APP-CD74 axis, a key communication pathway between neutrophils and endothelial cells. This suggests that the pathway may exacerbate immune activation and local atrial inflammatory responses by enhancing the “recruitment signals” from endothelial cells to neutrophils. In contrast, the ITGB2-ICAM2 axis between neutrophils and endothelial cells was diminished in the AF group. Unlike controls, the interactions between lymphoid cells and mural cells as well as between lymphoid cells and neutrophils in AF were decreased (Figure 7C).

### Animal model validation

**FIGURE 8:**
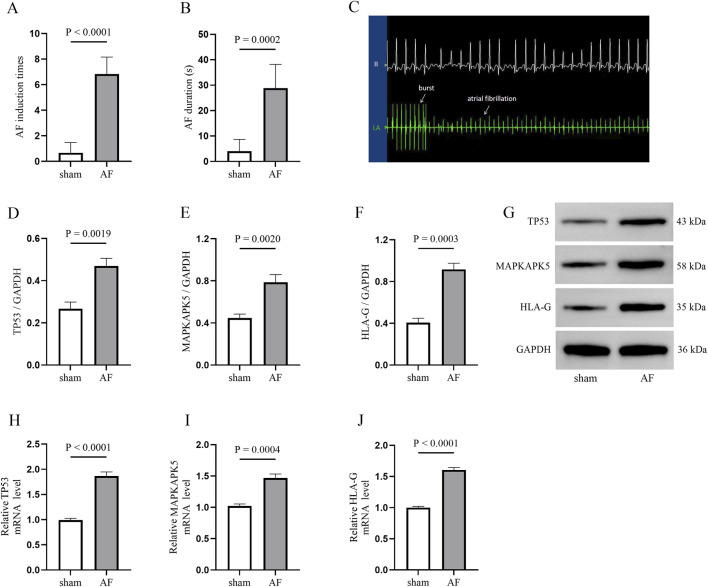
AF model validation in canines **(A,B)** The electrophysiological results of the AF induction **(A)** and duration **(B,C)** electrocardiographic (ECG) image illustrating AF induction **(D–G)** Western blotting image and quantative results for detecting three biomarkers expression between atriums from sham and AF groups **(H–J)** quantitative real-time polymerase chain reaction (qRT-PCR) results.

AF model was established by rapid atrial pacing in canines to further investigate the expression of TP53, MAPKAPK5 and HLA-G in atria. As shown in Figures 8A,B, electrophysiological measurements demonstrated that both the induction times and duration of AF were significantly higher in the AF canine model. Consistent with these findings, electrocardiographic (ECG) recordings revealed characteristic changes in the AF model (Figure 8C). Subsequently, Western blotting (Figures 8D–G) and qRT-PCR (Figures 8H–J) were employed to ascertain the expression levels of TP53, MAPKAPK5, and HLA-G in both the AF group and the sham group. The findings demonstrated that TP53, MAPKAPK5, and HLA-G were markedly overexpressed in the canine model of AF. The alignment between transcriptional and protein levels enhances our assurance in the differential expression of these genes in the etiology of AF.

## Discussion

MAMs are critical hubs for interorganelle communication, regulating calcium homeostasis, mitochondrial dynamics, and ER stress—processes that are highly relevant to atrial remodeling and arrhythmogenesis (Patergnani et al., 2011; Mohan and Talwar, 2025). Atrial remodeling, central to AF initiation and maintenance, involves electrical/structural changes like altered myocyte electrophysiology, apoptosis, and fibrosis, thereby facilitating the induction and duration of AF. Calcium imbalance triggers AF via afterdepolarizations. Subsequently, rapid irregular ventricular rates overwhelm myocardial calcium regulatory mechanisms, causing ER calcium leakage, accelerated fibrosis, and heterogeneous electrical conduction—fostering reentrant circuits that sustain AF (Guo Shuang et al., 2024). ER-mitochondria contacts (ERMCs), including MAMs, also underpin myocardial physiology. ER-mitochondrial calcium signaling is vital for contraction and energy metabolism in cardiomyocytes. ERMC abnormalities (e.g., ER stress, mitochondrial dysfunction) disrupt metabolism, induce calcium overload and mitochondrial apoptosis, promoting cardiomyocyte death and cardiomyopathy (Wang et al., 2021). Several studies have demonstrated the role of MAMs-related proteins in cardiovascular diseases (Chen et al., 2022; He et al., 2025); however, no research has reported the association between MAMs-related proteins and AF. Our identification of TP53, MAPKAPK5, and HLA-G as MAMs-related biomarkers provides novel insights into how MAM dysfunction contributes to AF pathogenesis, particularly the intersection of MAM dysfunction, immune dysregulation, and atrial remodeling.

TP53, a crucial regulator of cellular stress responses, exerts its effects via p53, which participates in cell cycle regulation, DNA repair, apoptosis, and cellular senescence. Notably, p53 modulates mitochondrial dynamics at MAMs through interactions with key mediators: it influences OPA1 (a core mediator of mitochondrial fusion and cristae structure) via Bak/Bax and OMA1, representing a mechanism linked to mitochondrial dysfunction and apoptosis in cardiomyopathies that may extend to atrial myocytes in AF (Zhang et al., 2022). Additionally, p53-induced mitochondrial fusion promotes cellular senescence by impairing mitochondrial function, as seen in vascular smooth muscle cell calcification—paralleling senescence-related atrial remodeling in AF (Phadwal et al., 2023). Previous studies have demonstrated that TP53 expression is correlates with AF severity, with activation associated with increased atrial fibrosis (Jesel et al., 2019). Moreover, an animal study has confirmed that inhibiting the p53 pathway can suppress cardiac fibroblast activation and mitigate the progression of myocardial fibrosis following myocardial infarction (Tamaki et al., 2013). Additionally, p53 enhances atrial inflammation, damaging cardiomyocytes and further driving atrial remodeling to form a vicious cycle in AF (Rennison et al., 2021), aligning with its association with immune-related pathways in our GSEA. It also regulates endogenous metabolites such as BNIP3 and BNIP3L, which mediate mitophagy—linking p53 to metabolic regulation in AF pathogenesis (Zhang et al., 2022). Collectively, TP53 integrates MAM-mediated mitochondrial dynamics, senescence, and mitophagy with fibrotic and inflammatory mechanisms, driving atrial remodeling and arrhythmogenesis in AF.

MAPKAPK5, a key protein kinase in the mitogen-activated protein kinase (MAPK) signaling pathway, mediates intracellular signal transduction, cellular stress responses, cell growth, and apoptosis. While its direct association with AF remains understudied, its downstream MAPK pathways are critical in AF pathogenesis—consistent with our GSEA results linking MAPKAPK5 to immune- and metabolism-related pathways, including MAPK signaling. Notably, MAPKAPK5 may influence AF via MAM-dependent mechanisms: Studies show that targeting MAPK pathways mitigates atrial remodeling: SMLC inhibits oxidative stress through the MsrA/p38 MAPK axis, protecting against atrial-ventricular remodeling in AF (Xu et al., 2024); α-BTX, an α7nAChR antagonist, suppresses the oxi-CaMKII/MAPK/AP-1 pathway to normalize calcium handling, alleviating mitochondrial dysfunction, apoptosis, and atrial fibrosis in AF models (Zhao et al., 2023). Our previous research also demonstrated that inhibiting the MAPK pathway can suppress cardiac macrophage pro-inflammatory polarization and fibroblast activation in canine AF, lowering atrial inflammation, improving atrial fibrosis, and delaying AF progression (He et al., 2021; Wang et al., 2022). Collectively, these findings suggest MAPKAPK5 contributes to AF by disrupting MAM-mediated calcium homeostasis, promoting mitochondrial dysfunction via oxidative stress, and amplifying inflammatory signaling—ultimately driving atrial structural/electrical remodeling and arrhythmogenesis.

HLA-G, a non-classical human leukocyte antigen (HLA) molecule, primarily functions in immune regulation, with well-established roles in tumor immunity, pregnancy, and transplant immune tolerance (Contini et al., 2020). Studies have indicated a genetically predicted causal relationship between an increase in the number of peripheral immune cells and the occurrence of AF (Feng et al., 2022). HLA-G lacks direct links to AF but may contribute to AF pathogenesis via MAM-mediated mechanisms—aligning with our GSEA results associating it with immune and calcium-related pathways: Mechanistically, soluble HLA-G (sHLA-G) interacts with CD8^+^ T cells via the CD8 coreceptor, triggering Fas/Fas-ligand-mediated apoptosis and inducing extracellular calcium influx (Puppo et al., 2002). Given MAMs’ role in calcium homeostasis, HLA-G may disrupt MAM-mediated calcium exchange in atrial myocytes, promoting calcium overload, abnormal electrical activity, and arrhythmogenesis. Immunologically, HLA-G-ILT2 interactions exert immunosuppressive effects by expanding MDSCs, which exacerbate atrial inflammation and fibrosis—hallmarks of atrial remodeling in AF (Zhang et al., 2008; Yang et al., 2020; Lin and Yan, 2021). Our immune infiltration data reinforce this link: MDSCs are highly infiltrated in AF, and HLA-G expression strongly correlates with MDSC abundance (cor >0.95), suggesting a functional axis where HLA-G promotes MDSC accumulation in atrial tissue to drive fibrotic remodeling and AF persistence. Additionally, HLA-G-expressing CD4^+^ T cells also regulate adaptive immunity, potentially linking to CD4^+^ lymphocyte changes in our AF patients (Pankratz et al., 2014). Collectively, these findings position HLA-G as a functional mediator linking MAM dysfunction (via calcium imbalance) to immune dysregulation, thereby driving atrial structural and electrical remodeling in AF, rather than a passive bystander.

Growing attention focuses on the critical role of immune responses in AF pathogenesis, involving immune cell infiltration into atria, interactions with atrial myocytes, and secretion of chemokines/cytokines to regulate the cardiac microenvironment. Precisely modulating immunity to promote myocardial recovery is a key goal in cardioimmunology (Xiao et al., 2021). Our immune-related analysis showed that all three genes correlated significantly with immune cells exhibiting differential infiltration between AF and control groups, particularly monocytes, dendritic cells, neutrophils, MDSC, and macrophages (Shi et al., 2023). Notably, TP53 strongly correlated with monocytes, MAPKAPK5 with dendritic cells, and HLA-G with macrophages. Studies indicate excessive monocyte activation (especially enhanced migration) contributes to atrial structural remodeling and post-ablation AF recurrence (Suehiro et al., 2021), whereas the CD14^+^CD16^+^ monocyte subset may reduce AF susceptibility by inhibiting abnormal activation or maintaining immune homeostasis (Wang et al., 2024). Dendritic cell-mediated responses play a critical role in AF-Crohn’s disease crosstalk (Qiu et al., 2024). Additionally, AF induces pro-inflammatory polarization of cardiac macrophages, driving neutrophil recruitment and macrophage accumulation; inhibiting macrophage polarization/migration alleviates myocardial inflammation and prevents AF progression (He et al., 2021; Ke et al., 2025). Activated and central memory CD4^+^/CD8^+^ T cells also showed significant intergroup differences, reflecting concurrent effects of immune microenvironment abnormalities on multiple T cell subsets. Senescent CD8^+^ T cells (marked by CD28 loss) and their transition from persistently activated CD8^+^ T cells (Effros et al., 2005), along with CD28 loss in CD4^+^CD28^−^ T cells and reduced PD-1 positivity in CD4^+^ lymphocytes (Kounis et al., 2020; Chang et al., 2022), are linked to AF pathogenesis. These changes in senescent T cells provide context for understanding differential T cell subset expression (Kazem et al., 2020; Li et al., 2025), suggesting their involvement in AF via shared immune regulatory networks. Collectively, our findings indicate the three genes may influence AF’s immune microenvironment, underscoring immune regulation’s crucial role in AF pathogenesis.

Cell communication analysis revealed that the activity of the APP-CD74 pathway from endothelial cells to neutrophils was enhanced in the disease group, suggesting a potential association with immune activation (Qian et al., 2025). CD74 is a cell surface receptor for the cytokine MIF(Huang et al., 2025), and studies have demonstrated that MIF can activate the p44/p42 MAPK signaling pathway and promote chemokine release through the CD74 receptor, thereby inducing MIP-2 secretion and ultimately leading to the recruitment and aggregation of neutrophils (Pellegrino et al., 2024). As a key kinase in the MAPK signaling pathway, MAPKAPK5 may be involved in signal transduction of this pathway. Combined with the findings that immune cell infiltration (e.g., CD8^+^ T cells and neutrophils) is significantly increased in AF patients, and the high expression of biomarkers TP53 and MAPKAPK5 shows a strong positive correlation with immune cell infiltration, it is suggested that the APP-CD74 pathway may exacerbate local atrial inflammatory responses by enhancing the “recruitment signals” from endothelial cells to neutrophils, thereby participating in atrial remodeling. This mechanism may be associated with the functional regulation of MAMs in AF (e.g., calcium exchange and changes in mitochondrial dynamics).

Moreover, potential interactions between drugs and biomarkers were predicted using the DGIdb database. The binding capabilities of these drugs to the biomarkers were further validated through molecular docking experiments. Notably, Pifithrin-α, a regulatory drug targeting TP53, has demonstrated its myocardial protective effects and its role in treating heart failure (Shao et al., 2021). However, there is no direct research on the application of TP53-related drugs in the AF treatment. Simvastatin, a commonly utilized statin, is primarily prescribed for lowering cholesterol and preventing cardiovascular diseases. Research have indicated that Simvastatin may have potential effects in the prevention and treatment of AF, likely due to its anti-inflammatory and antioxidative stress properties (Shiroshita-Takeshita et al., 2004; Cho et al., 2014). Our study showed an interaction between HLA-G and Simvastatin, suggesting that the drug may influence specific immune responses that are implicated in the pathogenesis of AF. However, the specific mechanisms underlying this interaction require further investigation.

In summary, our study identified TP53, MAPKAPK5, and HLA-G as MAMs-related biomarkers for AF and elucidated their potential mechanisms through a multi-dimensional analysis, providing new targets and insights for the prevention and treatment of AF. Noting that the limited sample size may restrict the generalizability of our findings, and larger, multi-center cohorts are needed for validation. In subsequent studies, we will further excavate the potential of these biomarkers and corresponding drugs through functional experiments and clinical trials. Moreover, MR analyses in this study were based on data from European populations. Although various GWAS databases offer data from diverse populations, the scarcity of high-quality GWAS data and the limited sample sizes in non-European populations impede the identification of crucial genetic variants, potentially compromising the accuracy of cross-ethnic result extrapolation. Future research should concentrate on Asian-ancestry-specific data collection and cross-ethnic comparisons to enhance the reliability of results.

## Data Availability

The original contributions presented in the study are included in the article/Supplementary Material, further inquiries can be directed to the corresponding author.

## Supplementary material

SUPPLEMENTARY FIGURE 1 Quality control (QC) of single-cell dataset **(A)** Distribution of cells in each sample after QC in terms of nFeature_RNA, nCount_RNA, and percent of mitochondrial genes **(B)** Screening of 2,000 highly variable genes **(C)** principal component analysis (PCA) dimensionality reduction analysis.

SUPPLEMENTARY FIGURE 2 Cell communication analysis targeting interaction between neutrophils and endothelial cells **(A,B)** Cellular communication network illustrating strength of interactions of neutrophils/endothelial cells: control **(A)** case **(B)**. **(C)** Key ligand-receptor interactions between defined cell-type pairs.

SUPPLEMENTARY TABLE 1 Clinical data in GSE14975 and GSE41177 datasets.

SUPPLEMENTARY TABLE 2 List of GO items and KEGG pathways enriched of AF-MAMRGs.

SUPPLEMENTARY TABLE 3 List of KEGG pathways enriched through GSEA and list of HALLMARK pathways enriched through GSVA.
