# Supplementary figures and images for: Integrated bioinformatic analysis reveals the underlying mitochondria-associated endoplasmic reticulum membranes-related biomarkers for atrial fibrillation
Source: Front Physiol. 2025 Sep 9;16:1647275. doi: 10.3389/fphys.2025.1647275 (PMC12454065; doi:10.3389/fphys.2025.1647275)

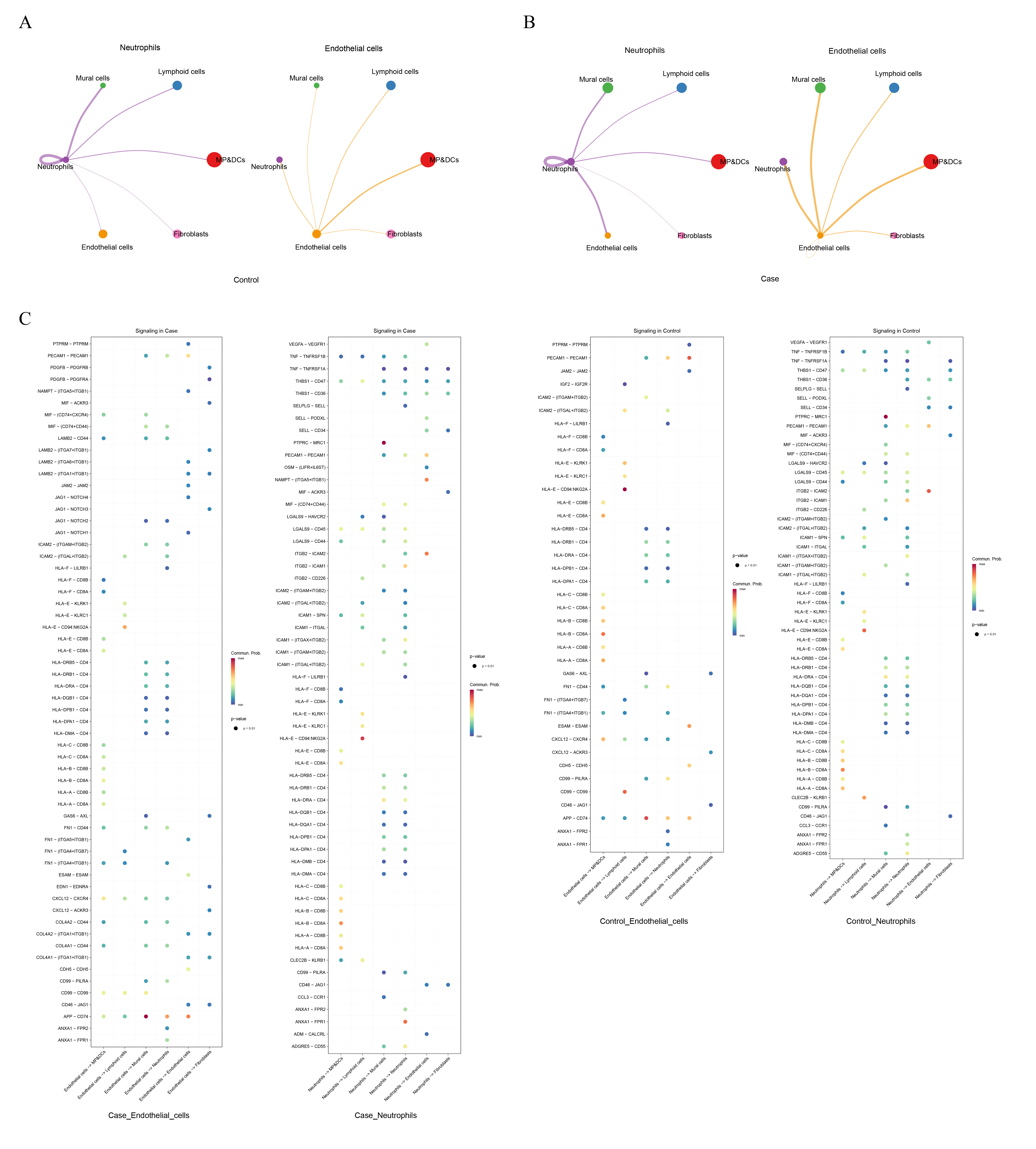

Supplement: Supplementary file 3 [file Image2.tif]

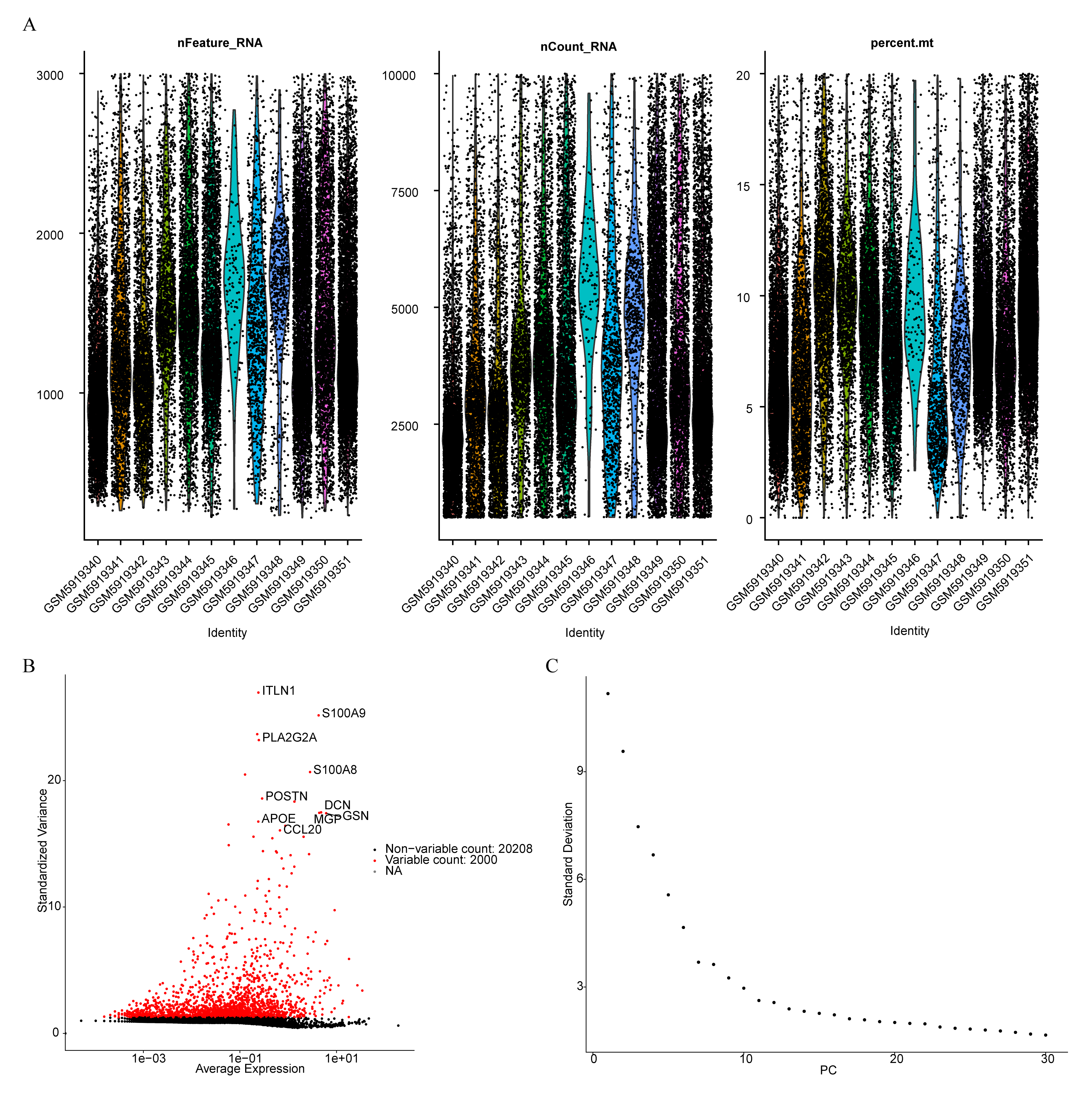

Supplement: Supplementary file 4 [file Image1.tif]
